# Supplementary figures and images for: Clock gene-dependent glutamate dynamics in the bean bug brain regulate photoperiodic reproduction
Source: PLoS Biol. 2022 Sep 6;20(9):e3001734. doi: 10.1371/journal.pbio.3001734 (PMC9447885; doi:10.1371/journal.pbio.3001734)

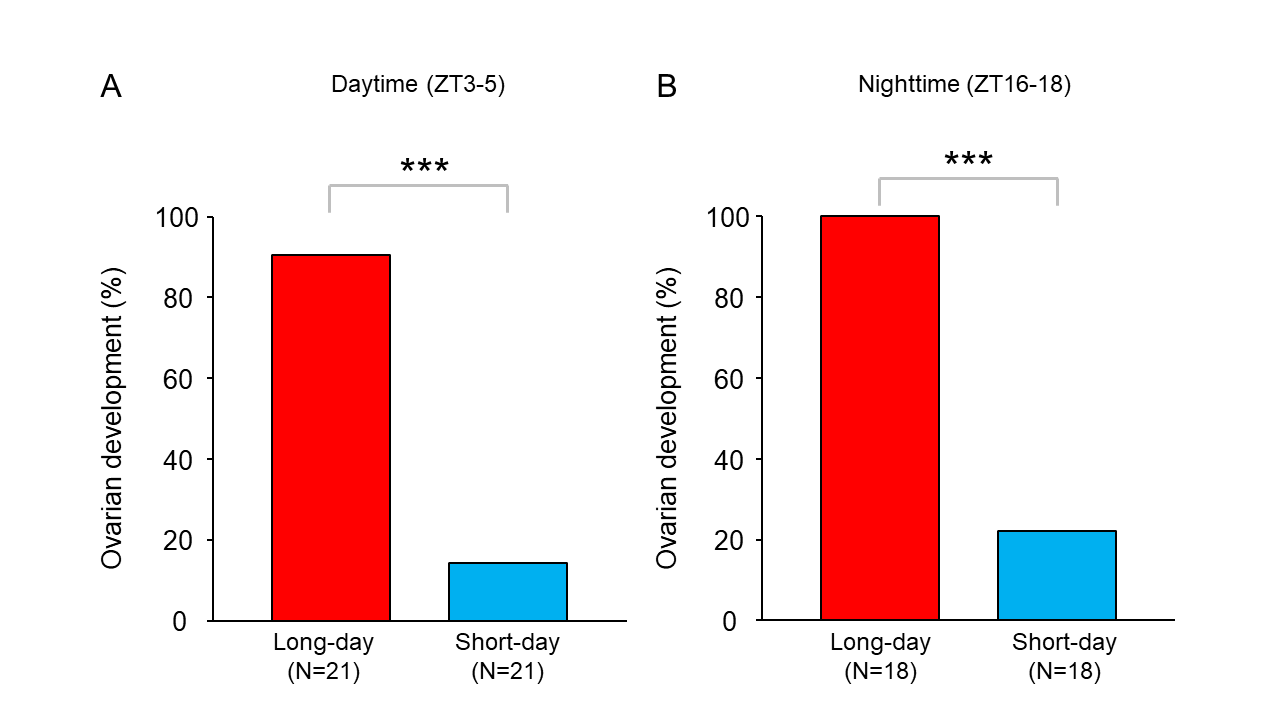

Supplement: S1 Fig — The rate of ovarian development was much higher under long-day conditions than under short-day conditions 20–22 days after eclosion in both daytime (A, zeitgeber time: ZT3–5) and nighttime (B, ZT16–18) dissection. χ2 test; *** P < 0.001. The underlying data can be found in the S1 Data datasheet of numerical values for each fig.xlsx. (TIF) [file pbio.3001734.s001.tif]

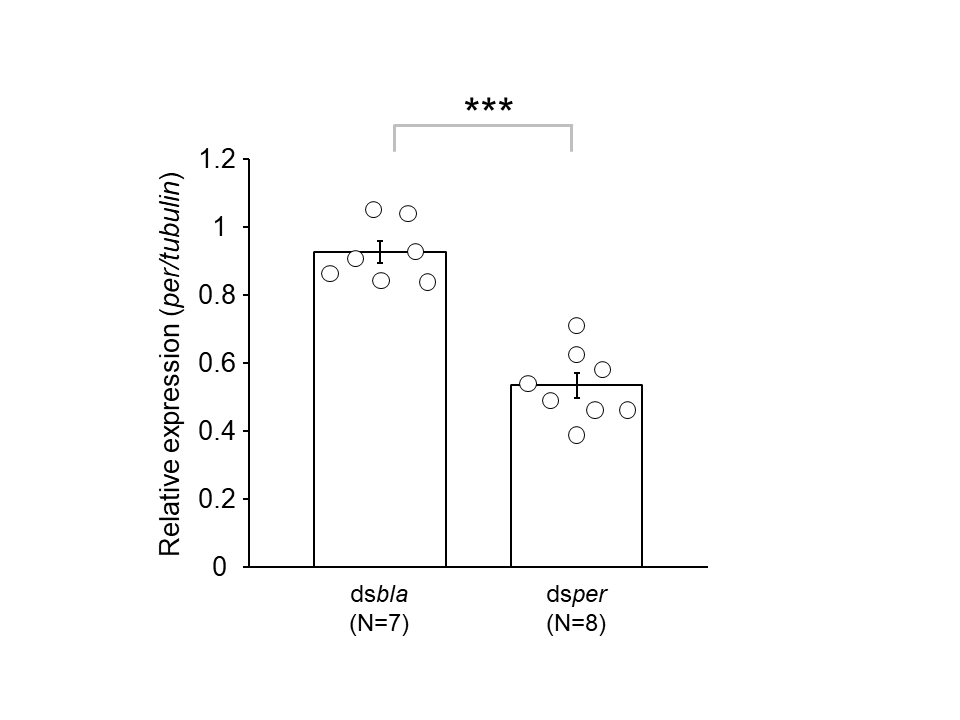

Supplement: S2 Fig — Columns with scatter plots show relative expression levels of period (per), which were normalized by expression of beta-tubulin (tubulin), in dsRNA for β-lactamase (dsbla) and dsRNA for per (dsper)-injected females. The relative expression levels of per were significantly lower in the dsper-injected group than in the control dsbla-injected group. Columns with error bars show mean ± SEM. Unpaired two-tailed t test, *** P < 0.001. The underlying data can be found in the S1 Data datasheet of numerical values for each fig.xlsx. (TIF) [file pbio.3001734.s002.tif]

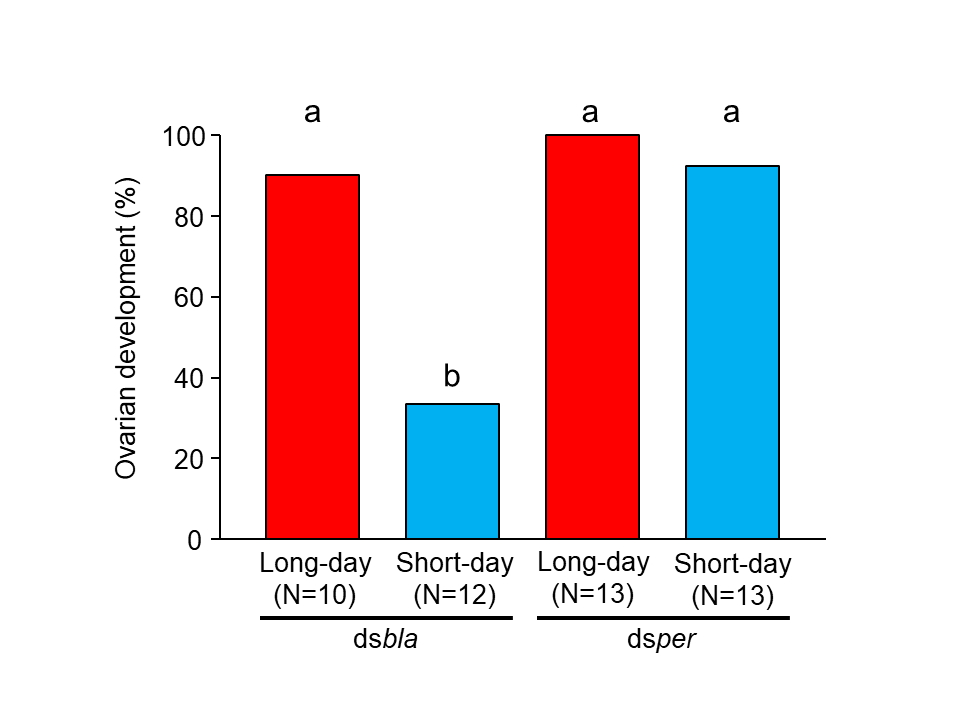

Supplement: S3 Fig — In control dsbla-injected females, the rate of ovarian development was much higher under long-day conditions than under short-day conditions. On the other hand, most dsper-injected females developed their ovaries even under short-day conditions, and there was no significant difference between long-day and short-day conditions within the dsper females. These dsRNA-injected females were used to measure brain extracellular glutamate concentration (Fig 1D), except for one dsbla-injected female under short-day conditions whose brain could not be successfully extracted. Columns with different letters show statistically significant differences (Tukey-type multiple comparisons for proportions, P < 0.05). The underlying data can be found in the S1 Data datasheet of numerical values for each fig.xlsx. (TIF) [file pbio.3001734.s003.tif]

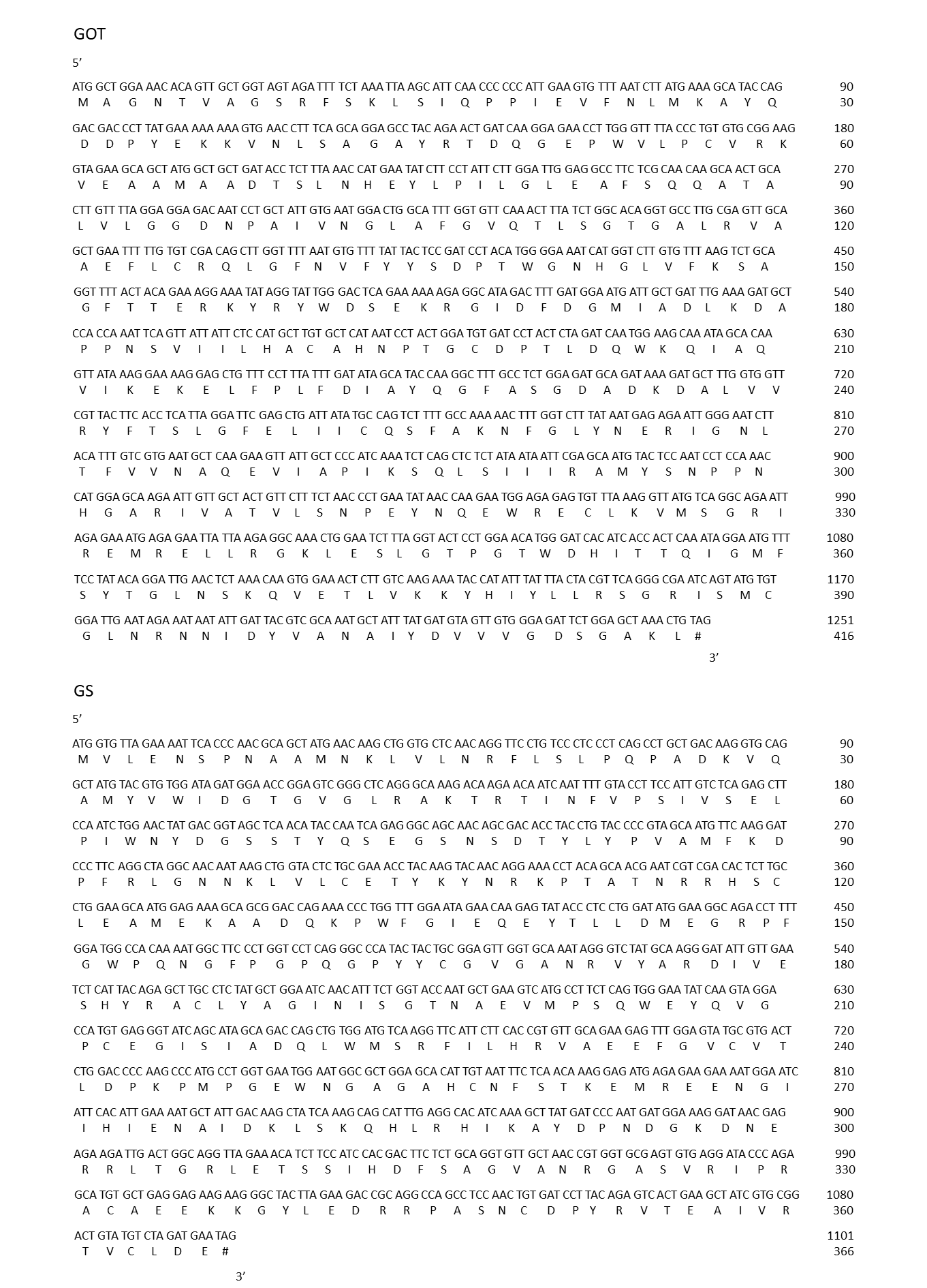

Supplement: S4 Fig — The symbol of # means a stop codon. (TIF) [file pbio.3001734.s004.tif]

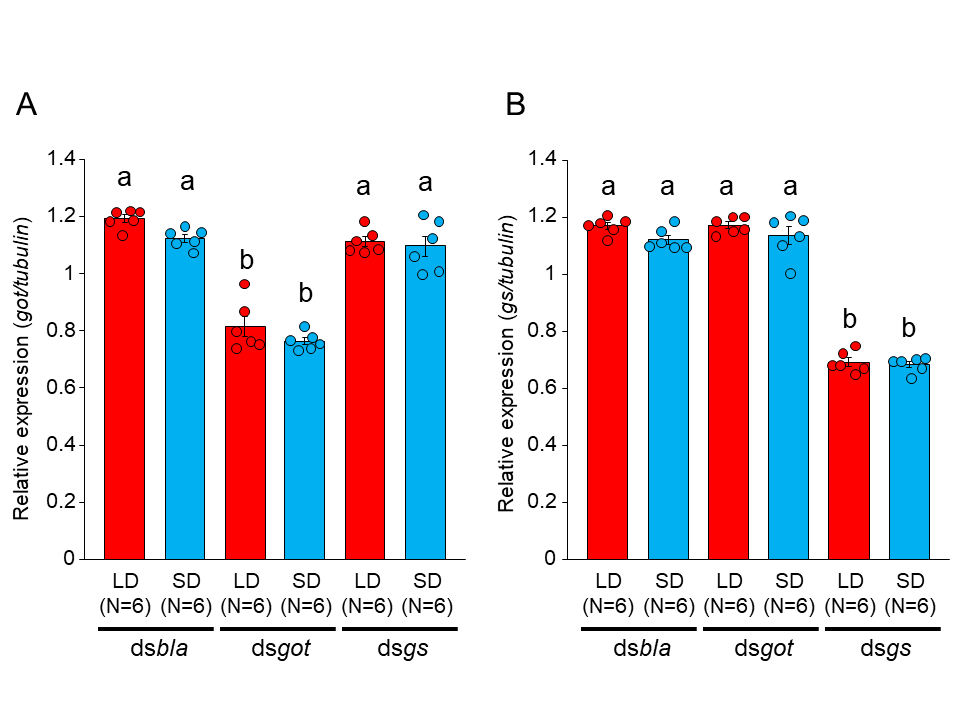

Supplement: S5 Fig — Columns with scatter plots show relative expression levels of got (A) and gs (B), which were normalized by expression of reference gene tubulin. dsRNA for got (dsgot) and that for gs (dsgs) specifically decreased the expression level of each target gene under both long-day (LD) and short-day (SD) conditions. Columns with error bars show mean ± SEM. Columns with different letters show statistically significant differences (Tukey–Kramer test, P < 0.001). The underlying data can be found in the S1 Data datasheet of numerical values for each fig.xlsx. (TIF) [file pbio.3001734.s005.tif]

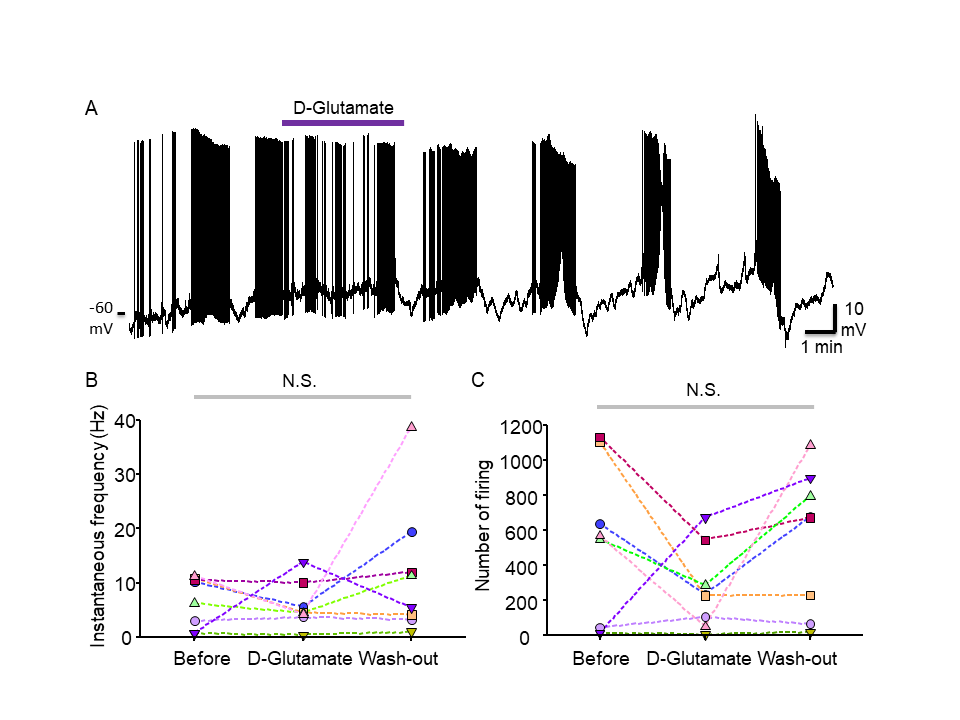

Supplement: S6 Fig — (A) A representative trace showing effects of 1 mM of D-Glutamate perfusion on the spontaneous firing activity of PI neurons. (B, C) Line graphs showing the (B) instantaneous frequency and (C) number of firing events in 3 min of “Before,” “D-Glutamate,” and “Wash-out” within each PI cell (n = 8). Perfusion of D-Glutamate did not have significant effects on the spontaneous neural activity of PI neurons, whereas slightly attenuated the neural activity in some cells. Steel–Dwass test, N.S.: not significant. The underlying data can be found in the S1 Data datasheet of numerical values for each fig.xlsx. (TIF) [file pbio.3001734.s006.tif]

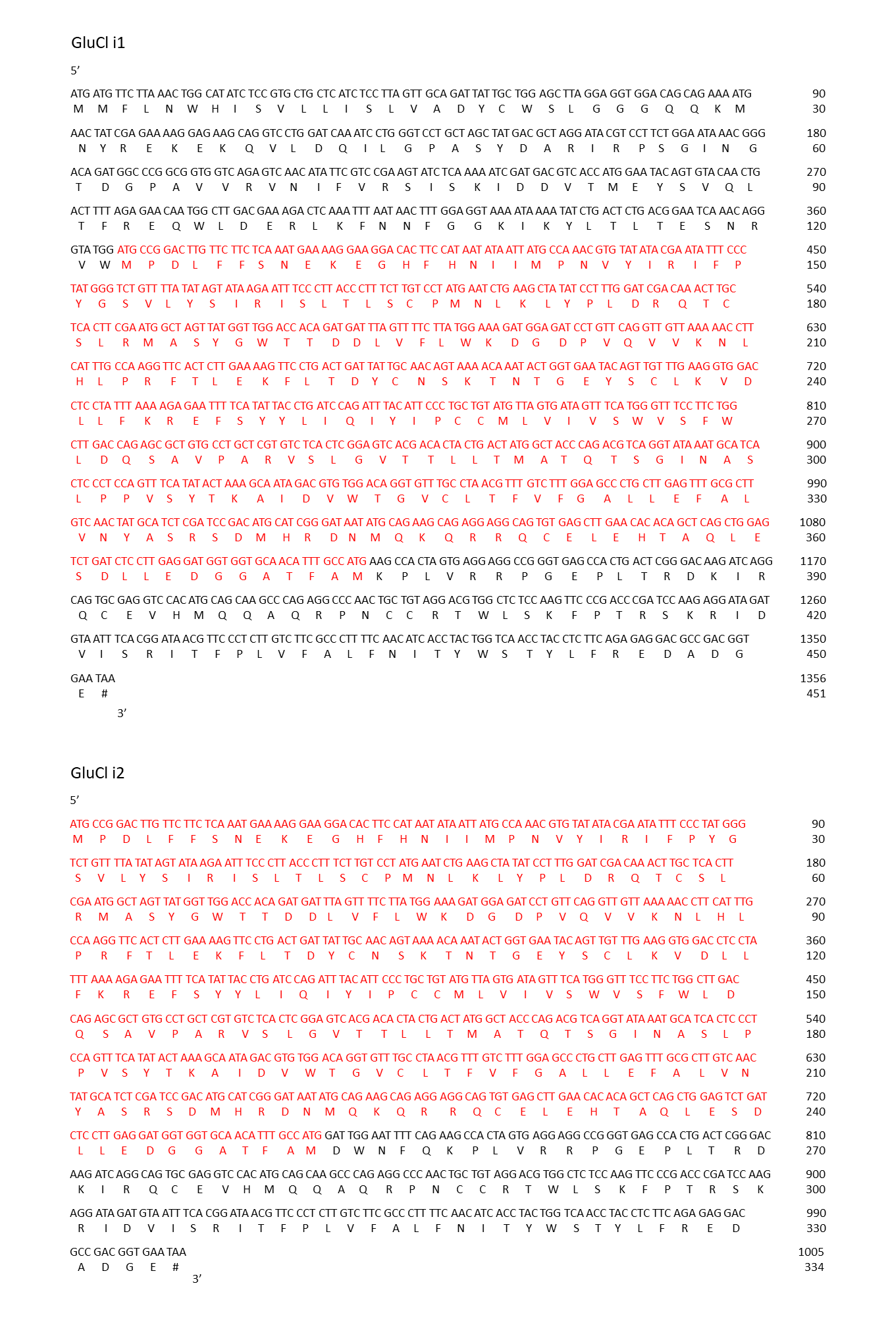

Supplement: S7 Fig — Red character sequences are the common sequence to the 2 isoforms. The symbol of # means a stop codon. (TIF) [file pbio.3001734.s007.tif]

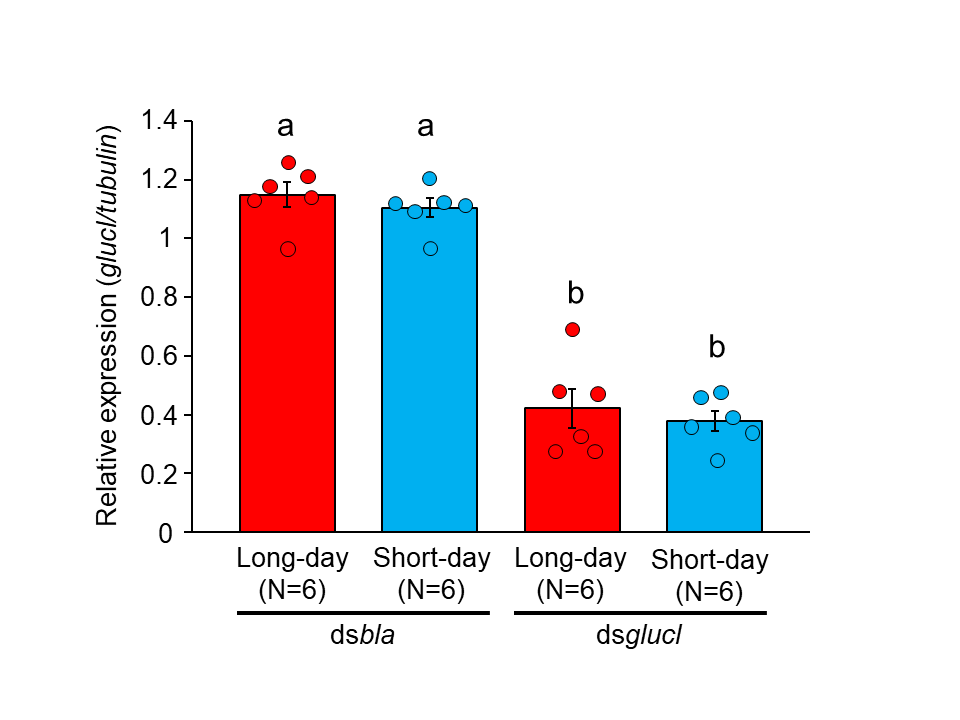

Supplement: S8 Fig — Columns with scatter plots show relative expression levels of glucl, which were normalized by the expression of reference gene tubulin. The relative expression levels of glucl were significantly lower in the dsRNA for glucl (dsglucl)-injected females than in the control dsbla-injected females under both long-day and short-day conditions. Columns with error bars show mean ± SEM. Columns with different letters show statistically significant differences (Tukey–Kramer test, P < 0.001). The underlying data can be found in the S1 Data datasheet of numerical values for each fig.xlsx. (TIF) [file pbio.3001734.s008.tif]

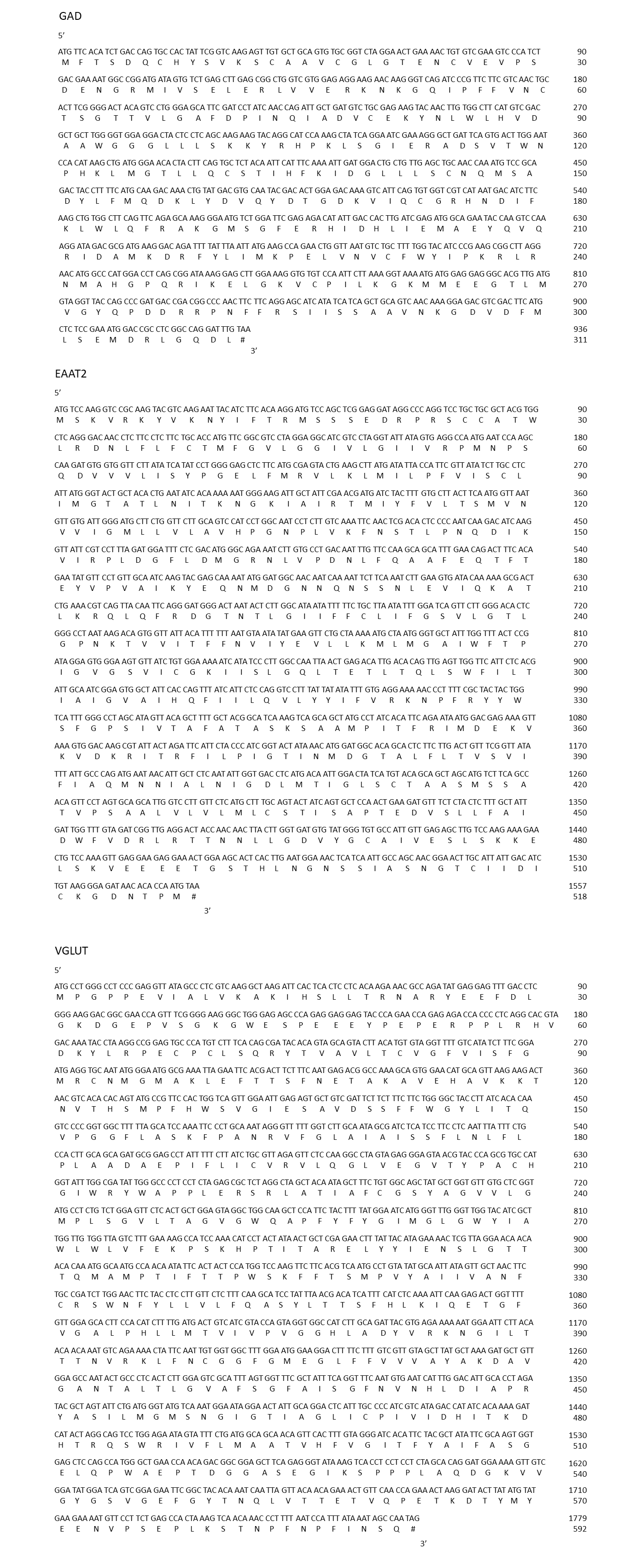

Supplement: S9 Fig — The symbol of # means a stop codon. (TIF) [file pbio.3001734.s009.tif]

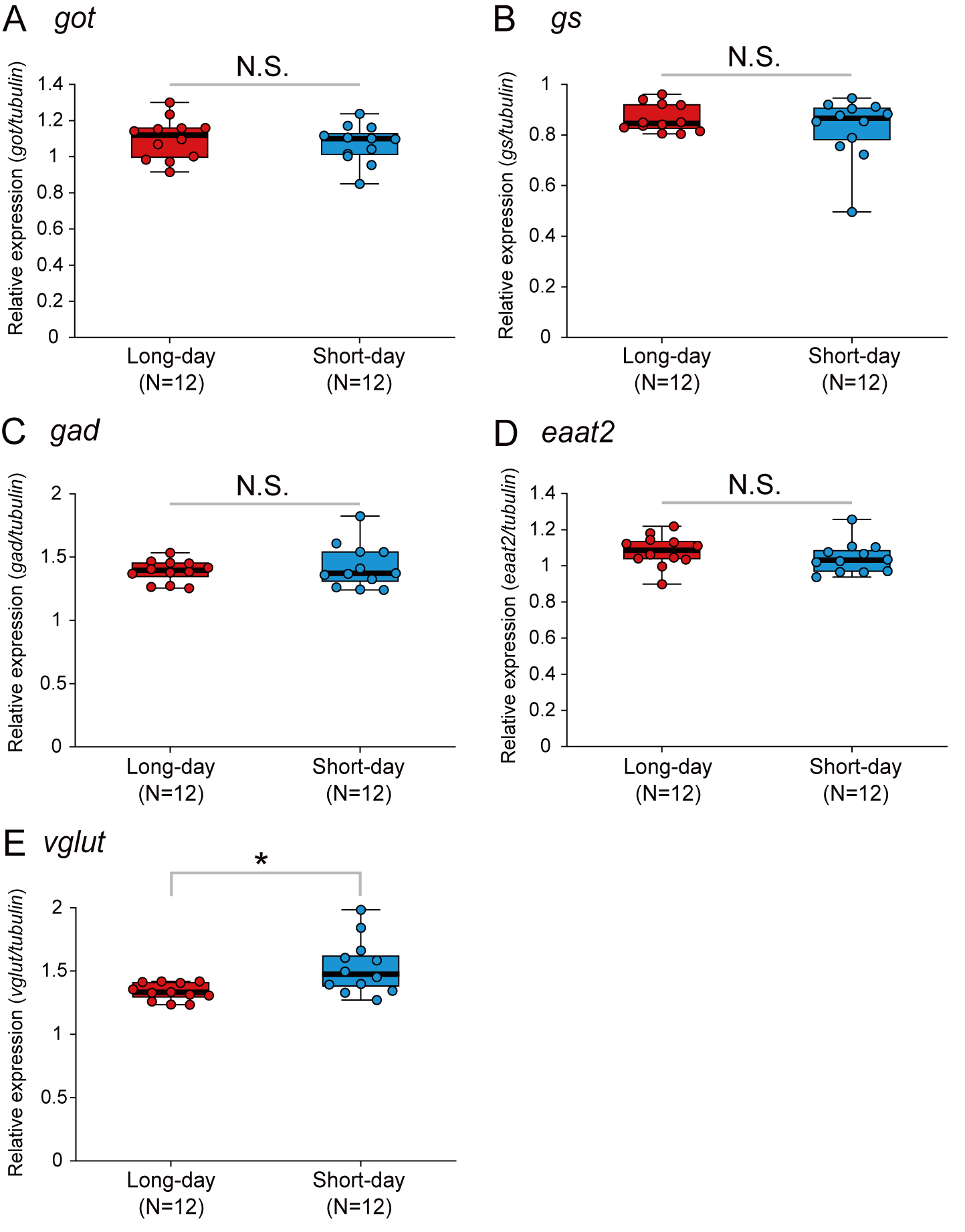

Supplement: S10 Fig — Box and scatter plots showing relative expression levels of (A) got, (B) gs, (C) gad, (D) eaat2, (E) vglut, which were normalized by expression of beta-tubulin (tubulin), in intact females under long days and short days. Lines at the top, middle, and bottom of the box plots indicate the upper quartile, median, and lower quartile, respectively. Upper and lower whiskers of the box plots indicate the maximum and minimum values, respectively. Two-tailed Mann–Whitney U test, * P < 0.05, N.S.: not significant. The underlying data can be found in the S1 Data datasheet of numerical values for each fig.xlsx. (TIF) [file pbio.3001734.s010.tif]

Fig6C

50 bp DNA ladder  
(NE-MWD50,  
Nippon Genetics)

*tubulin*

*gluc1*

500bp

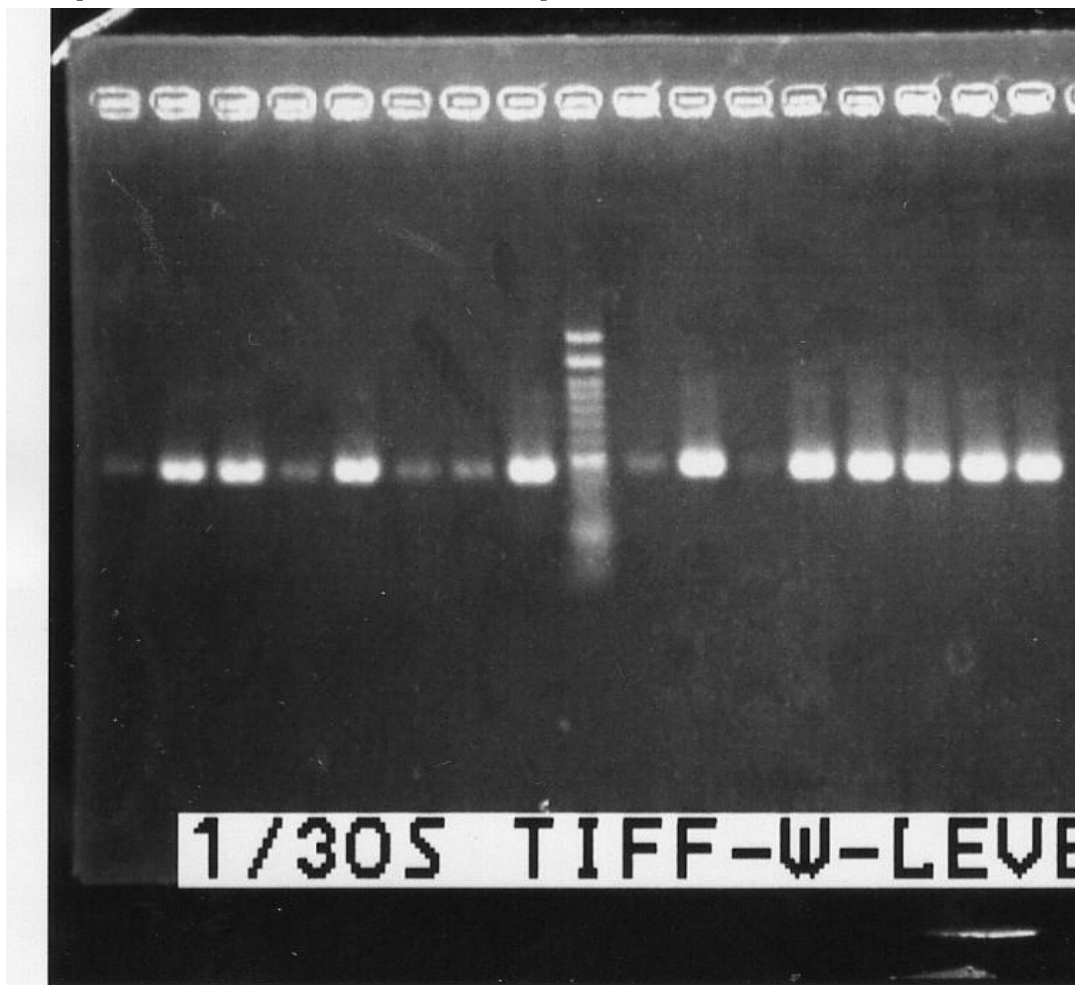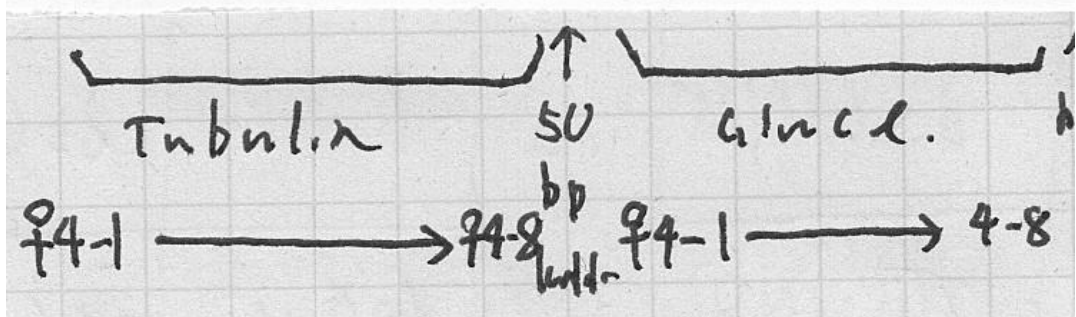

Supplement: S1 Raw image — (PDF) [file pbio.3001734.s014.pdf]
