# Supplementary material for: Clock gene-dependent glutamate dynamics in the bean bug brain regulate photoperiodic reproduction
Source: PLoS Biol. 2022 Sep 6;20(9):e3001734. doi: 10.1371/journal.pbio.3001734 (PMC9447885; doi:10.1371/journal.pbio.3001734)
Supplement: S1 Table — (DOCX) [file pbio.3001734.s011.docx]

| *tubulin* |  | Female | | | | |
| --- | --- | --- | --- | --- | --- | --- |
|  |  | a | b | c | d | e |
|  | cell 1 | + | ++ | + | + | + |
|  | cell 2 | ++ | ++ | + | ++ | ++ |
|  | cell 3 | ++ | ++ | + | ++ | ++ |
|  | cell 4 | ++ | + | ++ | + | ++ |
|  | cell 5 | + | + | + | ++ | ++ |
|  | cell 6 | + | ++ | ++ | + | + |
|  | cell 7 | + | + | + | + | ++ |
|  | cell 8 | + | + | ++ | ++ | ++ |
|  |  |  |  |  |  |  |
| *glucl* |  | Female | | | | |
|  |  | a | b | c | d | e |
|  | cell 1 | - | ± | + | + | ++ |
|  | cell 2 | - | + | + | ++ | ++ |
|  | cell 3 | + | + | - | + | + |
|  | cell 4 | + | + | ± | ++ | ++ |
|  | cell 5 | + | ++ | + | ++ | + |
|  | cell 6 | - | ++ | ++ | ++ | ++ |
|  | cell 7 | ± | + | + | ++ | ++ |
|  | cell 8 | ++ | + | ++ | ++ | ++ |
|  |  |  |  |  |  |  |
|  |  | ++ A strong band was found | | | |  |
|  |  | + A clear band was found | | | |  |
|  |  | ± A weak band was found | | | | |
|  |  | - There was no band | | |  |  |
