# Supplementary material for: Clock gene-dependent glutamate dynamics in the bean bug brain regulate photoperiodic reproduction
Source: PLoS Biol. 2022 Sep 6;20(9):e3001734. doi: 10.1371/journal.pbio.3001734 (PMC9447885; doi:10.1371/journal.pbio.3001734)
Supplement: S2 Table — (DOCX) [file pbio.3001734.s012.docx]

|  |  | Sequence (5' to 3') | Reference |
| --- | --- | --- | --- |
| For dsRNA synthesis | |  |  |
| *bla* | pGBetalacm-F1 | TCGCCGCATACACTATTCTC | Ikeno, et al., 2010 (1) |
|  | blaT7-Rv | TAATACGACTCACTATAGGTACGATACGGGAGGGCTTAC | Tamai, et al., 2019 (2) |
|  | blaT7-Fw | TAATACGACTCACTATAGGTCGCCGCATACACTATTCTC | Tamai, et al., 2019 (2) |
|  | pGBetalacm-R1 | TACGATACGGGAGGGCTTAC | Ikeno, et al., 2010 (1) |
| *per* | per10-F | GGGGAAGATTTCTCCCGTAG | Ikeno, et al., 2010 (1) |
|  | per21T7-R | TAATACGACTCACTATAGGGAACGTAGGGCATTTGCTGT | Ikeno, et al., 2010 (1) |
|  | per10T7-F | TAATACGACTCACTATAGGGGGGAAGATTTCTCCCGTAG | Ikeno, et al., 2010 (1) |
|  | per21-R | GAACGTAGGGCATTTGCTGT | Ikeno, et al., 2010 (1) |
| *got* | dsRNA Rp Got1 Fw | TTGTGAATGGACTGGCATTTGG |  |
|  | dsRNA Rp Got1 T7 Fw | TAATACGACTCACTATAGGTTGTGAATGGACTGGCATTTGG |  |
|  | dsRNA Rp Got1 Rv | AGAGAGCTGAGATTTGATGGGAG |  |
|  | dsRNA Rp Got1 T7 Rv | TAATACGACTCACTATAGGAGAGAGCTGAGATTTGATGGGAG |  |
| *gs* | dsRNA Rp GS2 Fw | CTCCTGGATATGGAAGGCAGAC |  |
|  | dsRNA Rp GS2 T7 Fw | TAATACGACTCACTATAGGCTCCTGGATATGGAAGGCAGAC |  |
|  | dsRNA Rp GS2 Rv | GGGTATCCTCACACTCGCAC |  |
|  | dsRNA Rp GS2 T7 Rv | TAATACGACTCACTATAGGGGGTATCCTCACACTCGCAC |  |
| *glucl* | Rp GluCl nest 1st Fw | TCCCTTACCCTTTCTTGTCCTATG |  |
|  | Rp GluCl nest 1st Fw T7 | TAATACGACTCACTATAGGTCCCTTACCCTTTCTTGTCCTATG |  |
|  | Rp GluCl nest 1st Rv | ACTGCCTCCTCTGCTTCTG |  |
|  | Rp GluCl nest 1st Rv T7 | TAATACGACTCACTATAGGACTGCCTCCTCTGCTTCTG |  |
|  |  |  |  |
| For qPCR |  |  |  |
| *tubulin* | tub4-F | CTGTCAACATGGTCCCATTCC | Ikeno, et al., 2008 (3) |
|  | tub4-R | GGAACAGTGAGGGCCCTGTA | Ikeno, et al., 2008 (3) |
| *per* | qPCR Rp per KD check2 Fw | AAGTGGAGGAAGTGCTGGTGAG | Hasebe and Shiga 2021 (4) |
|  | qPCR Rp per KD check2 Rv | CTGAACTTCCTCCGCTTCCACC | Hasebe and Shiga 2021 (4) |
| *got* | qPCR Rp Got1 Fw | ATACCAGGACGACCCTTATGAAA |  |
|  | qPCR Rp Got1 Rv | GCTTCTACCTTCCGCACACA |  |
| *gs* | qPCR Rp GS2 Fw | GTGCAGGCTATGTACGTGTGG |  |
|  | qPCR Rp GS2 Rv | CTGCCCTCTGATTGGTATGTTG |  |
| *glucl* | Rp GluCl qPCR Fw | GTGAATACAGTTGTTTGAAGGTGGA |  |
|  | Rp GluCl qPCR Rv | TGAGACACGAGCAGGCACA |  |
| *gad* | Rp GAD truth qpcr Fw | AGCATTCGATCCTATCAACCAGA |  |
|  | Rp GAD truth qpcr Rv | GTAGTCCTCCACCCCAAGCA |  |
| *eaat2* | Rp EAAT2 qPCR Fw | ACTTGGTGATGTGTATGGGTGTG |  |
|  | Rp EAAT2 qPCR Rv | AGTGCTTCCAGTTTCCTCTTCCT |  |
| *vglut* | Rp vGlut qpcr Fw | CGGCCAAAGCAGTGGAA |  |
|  | Rp vGlut qpcr Rv | TGATAAGGTAGCCCCAGAAGAAAG |  |
|  |  |  |  |
| For primary nested PCR | |  |  |
| *tubulin* | nest 1st tubulin Fw | CAAGATCAGAGAGGAATATCCCGAC | Hasebe and Shiga 2021 (4) |
|  | nest 1st tubulin Rv | ACTTCCTTCATTGACATTCGGCCTC | Hasebe and Shiga 2021 (4) |
| *glucl* | Rp GluCl nest 1st Fw | TCCCTTACCCTTTCTTGTCCTATG |  |
|  | Rp GluCl nest 1st Rv | ACTGCCTCCTCTGCTTCTG |  |
| For secondary nested PCR | |  |  |
| *tubulin* | nest 2nd tubulin Fw | CACATATTCAGTAGTCCCTTCACC | Hasebe and Shiga 2021 (4) |
|  | nest 2nd tubulin Rv | CTGCAACAGTAAGATAGCGACC | Hasebe and Shiga 2021 (4) |
| *glucl* | Rp GluCl nest 2nd Fw | TCCTTTGGATCGACAAACTTGCTC |  |
|  | Rp GluCl nest 2nd Rv | GTTGACAAGCGCAAACTCAAGC |  |

**References**

1. Ikeno T, Tanaka SI, Numata H, Goto SG. Photoperiodic diapause under the control of circadian clock genes in an insect. BMC Biol. 2010;8:116.

2. Tamai T, Shiga S, Goto SG. Roles of the circadian clock and endocrine regulator in the photoperiodic response of the brown-winged green bug *Plautia stali*. Physiological Entomology. 2019;44(1):43-52.

3. Ikeno T, Numata H, Goto SG. Molecular characterization of the circadian clock genes in the bean bug, *Riptortus pedestris*, and their expression patterns under long- and short-day conditions. Gene. 2008;419(1-2):56-61.

4. Hasebe M, Shiga S. Oviposition-promoting pars intercerebralis neurons show period-dependent photoperiodic changes in their firing activity in the bean bug. Proceedings of the National Academy of Sciences of the United States of America. 2021;118(9).
