# Supplementary material for: Clock gene-dependent glutamate dynamics in the bean bug brain regulate photoperiodic reproduction
Source: PLoS Biol. 2022 Sep 6;20(9):e3001734. doi: 10.1371/journal.pbio.3001734 (PMC9447885; doi:10.1371/journal.pbio.3001734)
Supplement: Attachment — Submitted filename: 220425 PLOS point-to-point_ss_MH final3.doc [file pbio.3001734.s015.doc]

The point-to-point responses to the reviewer’s comments are listed below.

The paragraphs starting with “#” denote our responses. In the “Revised Article with Changes Highlighted”, we highlighted with yellow all sentences in which changes have been made in response to the reviewers’ comments. Lines (***Lxxx~***) correspond to the number of lines in the clean main manuscript.

Reviewer 1

The manuscript by Hasebe and Shiga presents the surprising finding that bean bug whole brain glutamate efflux is sensitive to photoperiod and makes the case that this per gene dependent response acts via inhibitory glutamate receptors to alter neuronal excitability and reproductive behavior. In experimental systems such as Drosophila in which identified cells can be targeted emphasis has been on neuron connectivity and selective effects of neurotransmitters and neuropeptides in defined circuits. Here glutamate and gene expression are studied and manipulated at the whole brain level, which would usually be predicted to not be very informative. Thus, data supporting a role for a global change in glutamate in circadian behaviors and neuronal activity are surprising and interesting.

\# We are very grateful for encouraging comments to our research.

Major points:

1. First, a recurrent theme in the study is to compare data obtained from animals exposed to either "long-day" or "short-day" photoperiods. In many of the figures, the main point is that perturbing the system disrupts the differences seen in these two conditions. However, Figure 2A is presented differently than all of the other figures and the key comparison mentioned above is never made. If those differences are not significant in the control of Figure 2A, then the whole study is compromised. Furthermore, even when the presented statistical analysis shows that the difference is lost upon an RNAi treatment, the data still seem to show differences that are simply less dramatic. If paired tests were done for each manipulation, would differences between "long-day" or "short-day" photoperiods be significant? Could statistical insignificance simply reflect that irrelevant multiple comparisons were included that led p values to be inflated in a manner that does not relate to the　hypothesis being tested? By that view, photoperiodic changes are affected by glutamate, but the changes are not mediated by glutamate (i.e., as stated in the title).

\# We thank for the important suggestion. As reviewer suggested, only in the previous Figure 2A, we did not compare glutamate levels between different photoperiodic conditions. This was because we could not prepare a sufficient number of individuals for 3 dsRNA groups (ds*bla*, ds*got*, ds*gs*) under both long-days and short-days, so we measured glutamate levels of long day- and short day-groups at different times using different generations. Because it is not biologically appropriate to compare the results measured at different times using different generations, we did not compare long-day and short-day data in previous data of Figure 2A.

On the other hand, we fully agree with the reviewer's suggestion that it is essential for the subject of this paper to show that gene knockdown of glutamate enzymes (GOT and GS) disrupts the photoperiodic response of glutamate levels. Therefore, we prepared three dsRNA injection groups (ds*bla*, ds*got*, ds*gs*) under both long day and short day conditions in the same generation at the same time, and re-performed the measurement of glutamate levels. In the control ds*bla* group, the glutamate level was much higher under short day conditions than long day conditions similar to intact females (Fig.1). On the other hand, in the ds*got* injected group, the glutamate level was low under both long days and short days. In contrast, the glutamate level of ds*gs*-injected females was high in both long day and short day conditions. Both statistical methods (paired test between two photoperiod groups (not shown) and multiple comparison altogether) showed that there were no statistical differences in the glutamate levels between long days and short days within both ds*got* and ds*gs* groups. These strongly suggest that ds*got*, ds*gs*-injections abolish the photoperiodic change in glutamate level, which may induce loss of the photoperiodic response in reproductive functions.

We have replaced **Figure 2A** with a new graph based on the results of these re-experiments, and revised the main text ***L180-L187***.

2. Another issue is that the basis of the change in glutamate with photoperiod is unknown. Therefore, it remains possible that the whole brain data are correlative and not indicative of causation. In other words, it still remains possible that the real connection between glutamate and behavior arises at a few synapses (e.g., involving PDF neurons) and that those synapses are affected by the global perturbations used in the study. Even if the concerns presented in the previous paragraph were addressed, the data would be consistent with the authors' interpretation, but certainly the alternative has not been excluded. Given the concerns about presentation, interpretation and gaps in the understanding of the underlying biology, this manuscript does not seem currently acceptable.

\# We thank for the important comment. We agree with the reviewer’s comment; our results did not exclude the possibility that glutamate transmission at specific synapses is essential for the photoperiodic responses. Our previous manuscript would have leaded readers to misunderstand that photoperiodic glutamate change at a whole brain level is necessarily required for photoperiodic responses of reproduction. To be precise in our results, the specific glutamatergic signal in a pathway from clock cells to reproductive control cells is important for the photoperiodic responses in reproduction. The present photoperiodic change in whole brain-glutamate dynamics may be correlated with changes in glutamatergic transmission of this pathway.

We are also interested in the basis of the photoperiodic changes in glutamate. In the present study, we used ds*got* and ds*gs* as genetic tools to manipulate the extracellular glutamate levels. On the other hand, the mRNA levels of *got* and *gs* in the whole head did not significantly change according to photoperiod, suggesting that other glutamate-related factors are involved in changes in whole brain-glutamate dynamics. To access this point, we searched for glutamate-related genes that photoperiodically change their expression by quantitative polymerase chain reaction (qPCR) in the whole brain. Besides *got* and *gs*, we examined mRNA expressions of glutamate-converting enzyme, *glutamate decarboxylase* (*gad*), and glutamate transporters, *excitatory amino acid transporter 2* (*eaat2*) and *vesicular glutamate transporter* (*vglut*), which were reported to modulate the glutamate levels (1-3). We found that mRNA expression of *vglut* was significantly higher under short days than under long days correlated with extracellular glutamate levels, whereas other genes did not differ.

VGLUT transports glutamate to synaptic vesicles, and its genetic dysfunction significantly reduced extracellular glutamate release (3). In *R. pedestris,* another group recently reported that gene knockdown of *vglut* tended to abort the reproductive diapause (4). Thus, the photoperiodic change in *vglut* mRNA expression may be involved in the change in extracellular glutamate levels, which contributes to control of reproductive diapause. In rat neocortical circuits, the dynamic scaling of *vglut* mRNA expression occurs depending on the firing activity change of circuits, which may be involved in feedforward excitation and feedback inhibition of glutamate release (5, 6). Based on the report, we currently hypothesize that the glutamatergic circuits from clock cells to reproductive control cells change their firing activity according to photoperiod, which photoperiodically change the *vglut* expression for feedforward/feedback regulation of glutamate release.

We added the qPCR results of glutamate-related genes in the main text ***L301-L317, L978-982*** and made new **Figure 8**. We also carefully revised the discussion in the main text ***L335-L352,***

Minor points:

1. 12 hour light:12 hour dark conditions are not considered short-day. In the circadian field, short-day conditions would be in the winter when most of the day is dark.

\# We thank for the helpful comment. By convention, in insect photoperiodism studies, day length shorter than the critical day length are often referred to as short days. In our bug, the photoperiodic critical day length for reproduction is about 13 hour-light (7). Therefore 12 h light is a short day. To avoid confusion, we would like to call 12L12D as short-day conditions along with past studies.

2. Line 210, suggest changing firing rate to integrated activity.

\# We were sorry, but we could not find “firing rate” within the manuscript.

3. Line 223, suggest changing receives to binds.

\# Thanks. According to the reviewer’s comment, we corrected “GluCl receives glutamate” to “glutamate binds to GluCl” in ***L243.***

4. Lines 232 and 240, suggest changing inverting potential to reversal potential.

\# Thanks. According to the reviewer’s comment, we corrected “inverting potential” to “reversal potential” in ***L252.***

5. Line 294, suggest changing whereas to however.

\# Thanks. This text was deleted due to an additional qPCR experiment on glutamate-related genes.

6. Line 298, suggest changing is to could be.

\# Thanks. This text was also deleted due to an additional qPCR experiment on glutamate-related genes.

7. Throughout the manuscript, the text refers to brain glutamate release, but the discussion admits that changes could reflect changes in uptake. It would be better to make this admission when introducing the assay.

\# We are grateful for the helpful comment. Other reviewers have similar concerns, and the correct interpretation is that we measured “extracellular glutamate levels” not “glutamate release”. We have corrected "glutamate release" to "extracellular glutamate concentration/level".

Reviewer #2

In this manuscript Hasebe and Shiga examine the mechanisms underlying photoperiodic control of female reproductive diapause in the bean bug, Riptortus pedestris. The work is based on a striking induction of reproductive diapause by equinox conditions when compared to long day conditions (LD 16:8). The results are consistent with photoperiodic control of the levels of glutamate release, with higher levels of glutamate associated with equinox. The authors show that genetic manipulation of glutamate signaling disrupts induction of diapause by equinox. Interestingly, the authors show that injection RNA-interference constructs targeting the clock gene period disrupts photoperiodic differences in both glutamate levels and diapause, consistent with the central conclusions of the study that photoperiodic control of diapause is mediated by both the circadian clock and glutamate signaling. The relationships between the circadian system and photoperiodism are of great interest to the field as are mechanisms underlying such relationships. The work is therefore of significant interest. Addressing the following concerns would improve this promising work:

\# We are very grateful for encouraging comments to our research.

Major Concerns

1-1.) A major component of this study is the identification of glutamate as a neurochemical mediator of photoperiodism in the bean bug, but there are several aspects of the glutamate work that are cause for concern. First, the authors do not provide or cite evidence that the glutamate release assay is in fact a sensitive assay for release. Though it is certainly possible that this assay reflects relative amounts of glutamate being released by the explanted and desheathed brain, it is disconcerting that no evidence for this is present or cited, nor are alternative explanations for apparent photoperiodic differences discussed/eliminated.

It also seems possible that the assay doesn't reflect natural levels of release at all, but rather differences in the response of the brain to dissection. Might Ca2+ free media with high divalents, or some other intervention, be used to test if this assay really addresses "release?" Such conditions should prevent or drastically reduce release and result in much lower levels of GLU in the media than controls. If the authors cannot site evidence that their approach addresses release, they should provide such evidence. Showing that the other major neurotransmitters fail to show these photoperiodic differences would provide further support for the central model that glutamate release is modulated by photoperiod.

\# We thank for an important suggestion. As the reviewers mentioned, it is important to examine whether the measured extracellular glutamate in culture medium is physiologically released from brain cells or leaked from the brain due to injury to the brain. Thus, we additionally measured the extracellular glutamate concentration of brain cultured for 24 hours in the culture medium (MEM) supplemented with saline or tetrodotoxin (TTX), which is an inhibitor of Na^+^ channel and subsequently cause inhibition of the neurotransmitter release. Glutamate concentration in the MEM + TTX group was much lower (about 5 times) than that in the MEM + saline group. This result suggests that the measured extracellular glutamate is mainly due to neural activity-dependent release rather than leaking from the brain cells due to injury by manipulation. We added explanations of this additional result in ***L143-150*** and made new **Figure 1C**. On the other hand, other reviewers have similar concerns, and it remains possible that the measured glutamate contained a small amount of glutamate leaked from the brain. Thus, the correct interpretation is that we measured “extracellular glutamate levels” not “glutamate release”. We have corrected "glutamate release" to "extracellular glutamate concentration/level" throughout the manuscript.

We also thank for the suggestion about analysis of photoperiodic responses in other major neurotransmitters as negative controls. On the other hand, there is a possibility that measurement of other major neurotransmitters is not a negative control, because it has been reported that GABA (one of the major neurotransmitters) levels of in the brain-subesophageal ganglion change according to diapause-inducing conditions in the silkworm (8). We are also interested in changes in the dynamics of these other major neurotransmitters, but these studies need to be carefully examined, and are likely to deviate from the subject of this paper. Thus, we would like to just focus on glutamate dynamics in this paper.

1-2.) A critical alternative explanation, given the title and central conclusions of the study, is that there is a daily rhythm of glutamate abundance and that the phase of this rhythm is different between LD 12:12 and 16:8, thereby producing what looks like a difference in overall levels at the single time-point compared. It is well known that phases of entrainment differ between different photoperiods, but the authors have sampled brains to estimate glutamate concentrations at the same Zeitgeber Time for both conditions (ZT3-5). How can the authors be certain that the difference in glutamate concentration between short and long days is not simply a consequence of sampling out-of-phase glutamate release oscillations?

\# We are grateful for an important comment. As the reviewer mentioned, there was a possibility that the glutamate dynamics show daily oscillations, and the rhythm is just shifted depending on the day length conditions. Thus, to examine this point, we additionally performed the glutamate assay at a different time point (night time: ZT16-18). The glutamate levels were almost the same at daytime (ZT3-5) and nighttime (ZT16-18); glutamate concentration is about 160 µM under long-days and 280 µM under short-days both at daytime and nighttime. This result suggests that brain glutamate levels do not show drastic daily oscillations, and the photoperiodic change in glutamate may not be due to phase shifts in the daily rhythm.

We added explanations of this additional result in ***L139-141*** and made new **Figure 1B**.

2.) A strength of this study is that the authors have directly addressed the extent to which RNAi has resulted in a reduction in the expression levels of targeted genes. Based on the data confirming knockdown, it appears that such knockdowns were partial reductions in expression levels. In the case of GLU signaling manipulations, this makes sense, a complete loss of GLU signaling would certainly be fatal, and large reductions would be expected to have large and varied behavioral effects. The authors should comment on this. But more critically, it is not clear if the period knockdown described succeeded in abolishing circadian rhythms. Has the bug's clock been stopped by period RNAi? This could be determined by locomotor rhythms under constant condtions. It's possible that the partial knockdown of period mRNA does not stop the clock, which would make the relationship between circadian timekeeping and photoperiodic diapause induction less clear for this study. There are established post-transcriptional mechanisms in insect clocks that could support rhythmicity without normal levels of period mRNA or even mRNA rhythms. Thus, the relationship between the circadian clock and mechanisms of photoperiodic diapause induction are not as clear as suggested by the title of the manuscript, which claims that photoperiodic responses are mediated by circadian clock dependent glutamate dynamics.

\# We are grateful for the important suggestion. According to the reviewer’s suggestion, in ***L325-329*** of main text, we added the explanation that partial gene knockdown of GLU signaling by RNAi is effective because the complete loss of the GLU signal has a fatal effect.

As reviewer suggested, the effect of partial *period* knockdown on rhythmicity is an important point. Since the locomotor activity of *R. pedestris* is too weak to be measured, past studies have investigated the effect of *period* knockdown on the cuticle deposition rhythm that exhibited the major properties of circadian rhythms. Besides photoperiodic responses of reproduction, the cuticle deposition rhythm was abolished by the *period* knockdown in *R. pedestris* (9) (our present study used the same dsRNA of *period*). This result suggests that gene knockdown of *period* attenuates circadian clock functions important for circadian rhythm and photoperiodism. The result of previous study makes it easier for the reader to understand the relationship between the present partial *period* knockdown and the circadian rhythmicity in *R. pedestris*. Therefore, we added the explanation about the previous study in ***L151-154*** of main text.

The present study showed that the clock gene *period* is important for the photoperiodic change in glutamate dynamics. On the other hand, as reviewer is worried, we also understand that it is an overstatement to mention the involvement of circadian clock only by the knockdown of clock gene. Thus, we corrected the title "Circadian clock-dependent glutamate dynamics~" to "Clock gene-dependent glutamate dynamics~".

Other Concerns

a.) In Figure 3, the authors talk about firing patterns of neurons in the PI. The methods section says that electrophysiology experiments were done during the daytime (ZT00-12), but it is not clear if the results shown are average across multiple samples in the 12-h window or are they sampled in a different way. This needs some explanation in the text. Further both the got- and gs-RNAis seem to have the same effect on long- and short-day firing patterns of the PI neurons. Is this expected given how they are associated with reciprocal levels of glutamate release in short and long days? The authors should discuss this.

\# Thanks for the helpful comment. Our previous study showed that the PI neurons did not show a drastic daily change in their spontaneous activity (10). Thus, in this study, we recorded the neuronal activity of PI neurons from multiple samples across the daytime (ZT0-12). We added this explanation in ***L505-508*** of main text.

As reviewer mentioned, it is an important discussion point that ds*got* and ds*gs* have similar effects on PI spontaneous activity. ds*got* and ds*gs* adversely affected extracellular glutamate levels (ds*got* induced low glutamate, ds*gs* induced high glutamate levels). On the other hand, reproductive phenotypes and PI cell activities in ds*got* and ds*gs* groups seemed to be intermediate between those in the control ds*bla* under long-day and short-day conditions, whereas ds*got* and ds*gs* tended to induce the long day-, and short day-phenotypes, respectively. The previous study in the blow fly *Protophormia terraenovae* demonstrated that ablation of PDF-immunoreactive clock cells induced the rate of ovarian diapause to about 50% under both short- and long-day conditions, which may result from the inability of photoperiodic time measurement (11). Therefore, we currently assume that the glutamatergic signal is also involved in the photoperiodic time measurement in clock cells, and ds*got* and ds*gs* may have disrupt the photoperiodic time measurement, which induced the intermediate reproductive phenotypes and PI activities.

We added these discussions in ***L410-421*** of main text.

b.) In Figure 3B and 6D the relative firing proportions appear to have patterns that are different between the respective RNAis and photoperiods but one reason for not detecting statistical significance could be the use of chi-square goodness of fit tests (as inferred from the methods section). The N in these cases is too small for an accurate estimate from chi-square tests. Instead, if the authors use Fisher's exact tests, they may find statistical significance that may help their case.

\# We thank for suggestion. We used Tukey-type multiple comparisons for proportions in Figure 3B and 7D. Because application of two group-statistical tests (such as Fisher's test) without multi-group correction for multiple comparisons could often cause false positives, we would like to keep the result of Tukey-type multiple comparisons for proportions (with multi-group correction) in this study. We also performed Fisher's exact tests with Bonferroni correction or Holm–Bonferroni correction (for multiple comparisons). We found that the Fisher's exact tests with Bonferroni correction or Holm–Bonferroni correction gives the same result as the Tukey-type multiple comparisons for proportions in Figure 3B and 7D.

c.) The authors mention in the discussion (Line 357) that previous studies have shown that ablation of PI did not disrupt photoperiodic responses in the bean bug (Ref. 33). This is surprising given the focus here on photoperiodic changes in PI neuron firing activity and their known role in oviposition (Ref. 30). The authors should put this in context and explain why the electrophysiological properties of PI neurons is of interest here if they are not necessary for the phenomenon being examined.

\# Thanks. This study analyzed the glutamatergic signal as the circadian clock-driven neural signals that convey photoperiodic information to reproductive control cells. Since the photoperiodic change of glutamate can be detected at whole-brain levels, it is considered that the glutamatergic signal conveys photoperiodic information to multiple reproductive control cells. In *R. pedestris*, the oviposition-promoting PI neurons photoperiodically change their spontaneous neural activity depending on *period* expression, which suggests that PI neurons are one of strong candidates receiving photoperiodic information from the circadian clock (10). The PI neurons are only neurons that have been reported to exhibit a clock gene-dependent photoperiodic response at the level of neural activity. Thus, although the PI neurons only contribute to oviposition, we focused on PI neurons as one of the reproductive control cells that receive glutamatergic signals from clock cells. We added these explanations in ***L376-382*** of main text.

As we discussed, cells in the PL and their target CA are candidates for glutamate-mediated control of ovarian development. However, in insects, neurosecretory cells with various different characteristics are localized in the PL, and it has not been known which PL cells are important for ovarian development control in *R. pedestris*. In addition, juvenile hormones (JHs) secreted from CA are important for ovarian development in *R. pedestris* (12), suggesting PL cells control ovarian development via JH production in the CA. However, because of the difficulty for quantification of JH production or release in *R. pedestris,* the relevant cells of PL and CA have not been identified. In this reason, we chose large PI cells, which had been demonstrated to contribute to the promotion of oviposition through ILPs and DH44 release (10), as one of the downstream cells of the glutamate signal.

d.) There is no clarity on how statistics were performed in Fig. 4C-D. This is important for conclusions drawn in this section of the results. Were before, during perfusion, and wash-out values compared pairwise.? This would imply that there are three pairwise comparisons. It is not clear from the text if the p-values for each of these comparisons was 0.05 or was it adjusted for multiple comparisons. Also, it would be helpful if the line and symbols had some degree of transparency so that all the symbols can be clearly seen, because in some cases they overlap, and all 9 traces are not clearly visible.

\# Thank you for the helpful comment. We are sorry for the inadequate explanation of the statistical method in Fig. 4C-D. Since the data in Fig. 4C, D were not normally distributed, we first performed the Friedman test, which was a nonparametric pair-wise statistical test for multiple comparison (non-parametric version of ANOVA). The Friedman test showed that there was a significant difference among the 3 groups (before, during, wash-out) Thus, subsequently, we performed the *post-hoc* Steel-Dwass test (nonparametric multiple comparison test) for comparison between each group. The p-values in Figure 4C, D were calculated with the Steel-Dwass method (which includes multiple comparison corrections). We added the explanation of the multiple statistical comparison in ***L942-945*** of main text.

We also thank for the helpful comment of the Figure4C, D. According to reviewer’s comment, we increased transparency for overlapping circles and lines so that readers can see that there are multiple circles and lines.

e.) Line 324: Should be "Specific ablations targeting…"

\# Thanks. According to reviewer’s comment, we corrected “Specific ablations of targeting” to “Specific ablations targeting” in ***L364*** of main text.

Reviewer #3:

The overall goal of this study is to investigate the molecular and neuronal mechanisms underlying circadian clock-driven neural signals in the brain of the bean bug that convey photoperiodic information. Specifically, the authors are interested in the brain dynamics of a key neurotransmitter glutamate in different photoperiods and how glutaminergic signaling could be contributing to the photoperiodic response. The bean bug Riptortus pedestris is an ideal model for this research question given its unambiguous photoperiodism in reproductive phenotypes, such as ovary development and oviposition. The authors observed that glutamate release levels in the whole brain were significantly higher under SD conditions, which normally promote reproductive diapause. Interestingly, this photoperiod-dependent change in glutamate level is abolished when period is knocked down. Furthermore, the authors observed that the knock down of got and gs (2 glutamate metabolizing enzyme

genes) not only impact glutamate release level, but also abolished photoperiodic changes in reproductive phenotypes. The authors then leveraged electrophysiology to show that glutamate acts as an inhibitory signal to PI neurons via glutamate-gated chloride channel (GluCl), and RNAi knock down of GluCl, as well as got and gs can disrupt cellular photoperiodic responses of the PI neurons as well as reproductive diapause. In summary, the authors conclude that photoperiod regulates glutamate release in the brain of the bean bug, and the period gene is necessary to maintain this photoperiod response. They also show that photoperiodic-dependent change in glutamate dynamics plays an important role in photoperiodic control in reproductive physiology, and this is mediated via GluCl.

\# We are grateful for detailed interpretation of our results.

Major comments:

1. The authors need a negative control for their electrophysiology experiments in Figure 4. In addition to adding L-glutamate, they need to add something that the neurons do not respond to in order to show that the response they observed is specific to L-Glutamate. Perhaps D-Glutamate or a different amino acid?

\# We thank for the helpful suggestion. According to the reviewer’s suggestion, we additionally performed the D-Glutamate perfusion experiment. Perfusion of D-Glutamate did not have significant effects on the neural activity of PI neurons, whereas slightly attenuated the neural activity in some cells. This result suggests that the neural activity of PI cells is inhibited specifically by L-Glutamate. We added the explanation of the result in ***L236-239, L561-565*** of main text and made new **Figure S6**.

2. An additional negative control for the GluCl experiments would be the use of antagonists for GluCl. This would support that the inhibitory effects of glutamate is mediated by GluCl, and complement their genetic experiments. This is recommended but I do understand if the authors feel that their genetic experiment will suffice.

\# We are also grateful for the helpful comment. We fully agree with the reviewer’s suggestion, and additionally performed the co-perfusion of L-Glutamate and the antagonist for GluCl, picrotoxin (13, 14) by using voltage clamp method. We first perfused only L-Glutamate to the PI cell, and found that single perfusion of L-Glutamate evoked large outward currents at -20 mV. After then, we co-perfused L-Glutamate and picrotoxin, and found that the current induced by L-Glutamate was dramatically attenuated under picrotoxin After wash-out, the induction of outward current by single perfusion of L-Glutamate was restored to some extent. This result suggests that L-Glutamate acts on PI cells via GluCl. We added the explanation of the result in ***L264-271, L576-584,* *L954-961*** of main text and made new **Figure 6**.

3. There seems to be a disconnect in their model. Given that got and gs are not differentially expressed in LD vs SD, how is "period" changing glutamate concentration in the brain to achieve the difference they saw in LD vs SD. If got and gs are not the targets of clock control, the significance of their got and gs results is somewhat diminished. Could their enzyme activity be differentially expressed in LD vs SD, given that RNA expression is not equivalent to protein expression? Do the authors think they are normally involved in the photoperiodic response, or are they simply using them as "tools" to manipulate glutamate levels?

\# We are grateful for the important suggestion. As reviewer considered, we used ds*got* and ds*gs* as genetic tools to manipulate the extracellular glutamate levels. Indeed, ds*got* downregulated the extracellular glutamate levels, whereas ds*gs* upregulated. ds*got* and ds*gs* attenuated photoperiodic differences in extracellular glutamate levels (Please see new **Figure 2A**), and disrupted the photoperiodic responses in reproduction and PI neuronal activity. These results, using ds*got* and ds*gs* as a genetic manipulation tool for glutamate levels, clearly show important indications that extracellular glutamate levels are important for photoperiodic responses.

On the other hand, as reviewer suggested, the mRNA levels of *got* and *gs* in the whole head did not significantly change according to photoperiod. This suggests that other glutamate-related factors are involved in changes in glutamate dynamics. We are also interested in the basis of the photoperiodic changes in glutamate. To access this point, we searched for glutamate-related genes that photoperiodically change their expression in the whole brain by quantitative polymerase chain reaction (qPCR). Besides *got* and *gs*, we examined mRNA expressions of glutamate-converting enzyme, *glutamate decarboxylase* (*gad*), and glutamate transporters, *excitatory amino acid transporter 2* (*eaat2*) and *vesicular glutamate transporter* (*vglut*), which were reported to modulate the glutamate levels (1-3). We found that mRNA expression of *vglut* was significantly higher under short days than under long days correlated with extracellular glutamate levels, whereas other genes did not differ.

VGLUT transports glutamate to synaptic vesicles, and its genetic dysfunction significantly reduced extracellular glutamate release (3). In *R. pedestris,* another group recently reported that gene knockdown of *vglut* tended to abort the reproductive diapause (4). Thus, the photoperiodic change in *vglut* mRNA expression may be involved in the change in extracellular glutamate levels, which contributes to control of reproductive diapause. In rat neocortical circuits, the dynamic scaling of *vglut* mRNA expression occurs depending on the firing activity change of circuits, which may be involved in feedforward excitation and feedback inhibition of glutamate release (5, 6). Based on the report, we currently hypothesize that the glutamatergic circuits from clock cells to reproductive control cells change their firing activity according to photoperiod, which photoperiodically change the *vglut* expression for feedforward/feedback regulation of glutamate release.

We added the qPCR results of glutamate-related genes in the main text ***L301-L317*** and made new **Figure 8**. We also carefully revised the discussion in the main text ***L335-L352.***

4. Line 357: Given that ablation of PI did not disrupt photoperiodic response in bean bug, why did the authors choose to investigate the direct effects of glutamate on PI neuronal activity? The authors noted that a good candidate brain region for glutamate-mediated control of ovarian development if the PL or CA. Why did they not conduct their electrophysiology experiments on PL or CA?

\# Thanks. This study analyzed the glutamatergic signal as the circadian clock-driven neural signals that convey photoperiodic information to reproductive control cells. Since the photoperiodic change of glutamate can be detected at whole-brain levels, it is considered that the glutamatergic signal conveys photoperiodic information to multiple reproductive control cells. In *R. pedestris*, the oviposition-promoting PI neurons photoperiodically change their spontaneous neural activity depending on *period* expression, which suggests that PI neurons are one of strong candidates receiving photoperiodic information from the circadian clock (10). Although the PI neurons only contribute to promoting oviposition, but not ovarian development, they are only neurons that have been reported to exhibit a clock gene-dependent photoperiodic response at the level of neural activity. Thus, we focused on PI neurons as one of the reproductive control cells that receive glutamatergic signals from clock cells. We added these explanations in ***L376-382*** of main text.

As reviewer suggested, PL and CA are candidates for glutamate-mediated control of ovarian development. However, in insects, neurosecretory cells with various different characteristics are localized in the PL, and it has not been known which PL cells are important for ovarian development control in *R. pedestris*. In addition, juvenile hormones (JHs) secreted from CA are important for ovarian development in *R. pedestris* (12), suggesting PL cells control ovarian development via JH production in the CA. However, because of the difficulty for quantification of JH production or release in *R. pedestris,* the relevant cells of PL and CA have not been identified. In this reason, we chose large PI cells, which had been demonstrated to contribute to the promotion of oviposition through ILPs and DH44 release (10), as one of the downstream cells of the glutamate signal.

Minor comments:

1. The authors should insert data points onto all bar graphs when possible. I understand that this is not possible when they are plotting ovary development (%), but it is certainly possible in Figure 1B-C, Figure S2, S5, and S7.

\# We are grateful for the helpful comment. According to the comment, we added the scatter plotting to **Figure 1B-D, Figure S2, S5,** and **S8**.

2. Line 79: The authors should include more recent citations, e.g. https://pubmed.ncbi.nlm.nih.gov/31767753/

https://pubmed.ncbi.nlm.nih.gov/32541062/

\# We thank for the helpful comment. According to the comment, we added the recent citations in ***L82***.

3. Line 177: specify downregulated "only in SD".

\# Thanks. According to other reviewer’s suggestion, we re-examined the extracellular glutamate level in ds*bla, got, gs*, groups and revised the sentence of ***L180-187***.

4. Line 180: specify upregulated "only in LD".

\# Thanks. According to other reviewer’s suggestion, we re-examined the extracellular glutamate level in ds*bla, got, gs*, groups and revised the sentence ***L180-187***.

5. How did the authors determine the concentration of L-Glutamate to use in their experiments, e.g. did they do a dose-response experiment?

\# We thank for the comment. The previous electrophysiological study in the *Drosophila* large ventrolateral neurons showed that bath application of 1 mM glutamate had a sufficient effect on their neural activity via GluCl (15). Also in the *Xenopus* oocytes expressing GluCl by RNA injections, dose-dependent increase in glutamate induced currents became a plateau at about 1 mM (13). Thus, to investigate the sufficient effect of glutamate on PI cells, we determined to perfuse glutamate at a high concentration of 1 mM. We added the explanation in ***L548-554***.

6. Additional of a model figure would be really helpful to the readers.

\# We thank for the helpful suggestion. According to the reviewer’s suggestion, we made a new **Figure 9** showing a conceptual diagram summarizing the results of this research.

7. How homogeneous is the efficiency of glutamate release (into MEM)? Could this have contributed to the variability in Fig 2A?

\# We thank for the comment. As the reviewer was interested, there were some variations in extracellular glutamate levels (glutamate release) even under the same condition. We also think that the difference in efficiency of glutamate release may be one of factors for this variability. On the other hand, there are some possible factors for this variability: the difference in KD efficiency by dsRNA, biological diversity of organism under the same condition. Although we cannot determine which factor causes this variability, even in the presence of such variability, we can see the significant effects of photoperiod and RNAi, which suggesting their significance in control of glutamate levels. It is useful for the reader to clarify that the present comparisons were made in the presence of variations. Thus, as suggested in other reviewer’s comment, we added the scatter plotting to **Figure 1B-D, Figure S2, S5,** and **S8** for showing variations of KD efficiency and glutamate levels.

**Reference**

1.	Featherstone DE, Rushton E, Broadie K. Developmental regulation of glutamate receptor field size by nonvesicular glutamate release. Nature neuroscience. 2002;5(2):141-6.

2.	Rothstein JD, Dykes-Hoberg M, Pardo CA, Bristol LA, Jin L, Kuncl RW, et al. Knockout of glutamate transporters reveals a major role for astroglial transport in excitotoxicity and clearance of glutamate. Neuron. 1996;16(3):675-86.

3.	Wang DV, Viereckel T, Zell V, Konradsson-Geuken A, Broker CJ, Talishinsky A, et al. Disrupting Glutamate Co-transmission Does Not Affect Acquisition of Conditioned Behavior Reinforced by Dopamine Neuron Activation. Cell Rep. 2017;18(11):2584-91.

4.	Des Marteaux L, Xi J, Mano G, Goto SG. Circadian clock outputs regulating insect photoperiodism: A potential role for glutamate transporter. Biochem Biophys Res Commun. 2022;589:100-6.

5.	Erickson JD, De Gois S, Varoqui H, Schafer MK, Weihe E. Activity-dependent regulation of vesicular glutamate and GABA transporters: a means to scale quantal size. Neurochem Int. 2006;48(6-7):643-9.

6.	De Gois S, Schafer MK, Defamie N, Chen C, Ricci A, Weihe E, et al. Homeostatic scaling of vesicular glutamate and GABA transporter expression in rat neocortical circuits. The Journal of neuroscience : the official journal of the Society for Neuroscience. 2005;25(31):7121-33.

7.	Numata H, Hidaka T. Photoperiodic control of adult diapause in the bean bug, Riptortus clavatus Thunberg (Heteroptera: Coreidae) I. Reversible induction and termination of diapause. Applied Entomology and Zoology. 1982;17(4):530-8.

8.	Tsuchiya R, Kaneshima A, Kobayashi M, Yamazaki M, Takasu Y, Sezutsu H, et al. Maternal GABAergic and GnRH/corazonin pathway modulates egg diapause phenotype of the silkworm Bombyx mori. Proceedings of the National Academy of Sciences of the United States of America. 2021;118(1).

9.	Ikeno T, Tanaka SI, Numata H, Goto SG. Photoperiodic diapause under the control of circadian clock genes in an insect. BMC Biol. 2010;8:116.

10.	Hasebe M, Shiga S. Oviposition-promoting pars intercerebralis neurons show period-dependent photoperiodic changes in their firing activity in the bean bug. Proceedings of the National Academy of Sciences of the United States of America. 2021;118(9).

11.	Shiga S, Numata H. Roles of PER immunoreactive neurons in circadian rhythms and photoperiodism in the blow fly, Protophormia terraenovae. The Journal of experimental biology. 2009;212(Pt 6):867-77.

12.	Ando Y, Matsumoto K, Misaki K, Mano G, Shiga S, Numata H, et al. Juvenile hormone III skipped bisepoxide, not its stereoisomers, as a juvenile hormone of the bean bug Riptortus pedestris. General and comparative endocrinology. 2020;289:113394.

13.	Etter A, Cully DF, Liu KK, Reiss B, Vassilatis DK, Schaeffer JM, et al. Picrotoxin blockade of invertebrate glutamate-gated chloride channels: subunit dependence and evidence for binding within the pore. Journal of neurochemistry. 1999;72(1):318-26.

14.	Raymond V, Sattelle DB, Lapied B. Co-existence in DUM neurones of two GluCl channels that differ in their picrotoxin sensitivity. Neuroreport. 2000;11(12):2695-701.

15.	McCarthy EV, Wu Y, Decarvalho T, Brandt C, Cao G, Nitabach MN. Synchronized bilateral synaptic inputs to Drosophila melanogaster neuropeptidergic rest/arousal neurons. The Journal of neuroscience : the official journal of the Society for Neuroscience. 2011;31(22):8181-93.
