# Supplementary material for: Clock gene-dependent glutamate dynamics in the bean bug brain regulate photoperiodic reproduction
Source: PLoS Biol. 2022 Sep 6;20(9):e3001734. doi: 10.1371/journal.pbio.3001734 (PMC9447885; doi:10.1371/journal.pbio.3001734)
Supplement: Attachment — Submitted filename: 220628 PLOS 2nd point-to-point2.doc [file pbio.3001734.s016.doc]

The point-to-point responses to the editor’s and reviewer’s comments are listed below. The paragraphs starting with “#” denote our responses. We highlighted with yellow all sentences in which changes have been made in response to the reviewers’ comments in the text.

Editor

1. In the final round of peer review, two of the reviewers raised some concerns with the strength of the Fig 8 data examining vglut transporter mRNA expression changes with the photoperiod. Our editorial view is that this data should be taken out of the main manuscript given these concerns. We are happy to have you place these in the supplemental materials, revising the abstract and main body text and model accordingly to de-emphasize these results.

\# We thank for the helpful suggestion. We fully agree with the editor’s suggestion. According to the comment, we transferred the previous Fig. 8 data to supplemental data (S10 Fig.) and removed the abstract and result texts about *vglut* expression, and revised the discussion in ***L320-334*** and conceptual diagram (new Figure 8).

2. In addition, we'd ask that you consider a minor title change, along the lines of: Clock-gene-dependent glutamate dynamics in the bean bug brain regulate photoperiodic reproduction.

\# We thank for the helpful comment. According to the suggestion, we changed the title as “Clock-gene-dependent glutamate dynamics in the bean bug brain regulate photoperiodic reproduction”.

3. As you address these items, please take this last chance to review your reference list to ensure that it is complete and correct. If you have cited papers that have been retracted, please include the rationale for doing so in the manuscript text, or remove these references and replace them with relevant current references. Any changes to the reference list should be mentioned in the cover letter that accompanies your revised manuscript.

\# In relation to moving the data in previous Fig. 8 to supplementary data and revising the discussion, the reference list was partially revised. Please refer to "Revised Article with Changes Highlighted" for the revised part of the cited paper list. We also mentioned in the cover letter that accompanies our revised manuscript.

4. Regardless of the method selected, please ensure that you provide the individual numerical values that underlie the summary data displayed in the following figure panels as they are essential for readers to assess your analysis and to reproduce it:
Fig 1 B-D; Fig 2A-D; Fig 3B-D; Fig 4C-D; Fig 5C; Fig6B; Fig 7A-F (NB – Fig 8 as well if moved to SF), Supplemental Fig 1A-B; Fig 2; Fig 3, Fig 5A-B; Fig 6B-C; Fig 8

\# According to the suggestion, we added a datasheet of numerical values for each figure.

Reviewer 1

In this revision the authors show that glutamate release from the bean bug brain explant is affected by lighting schedule. Furthermore, this release is sensitive to TTX and period gene expression. Finally, this glutamate release is implicated in behavior by knocking down glutamate metabolizing enzymes and an inhibitory ionotropic glutamate receptor, which is shown to alter electrical activity in select neurons. Whether the effects of these global perturbations reflect a widespread mechanism or an effect on a few key clock neuron synapses remains to be determined. All of the above findings are convincing, but the manuscript closes with gene expression data in Figure 8 that are overinterpreted: the authors focus on a statistically significant change in vglut mRNA, but the effect is tiny. It is known that such a small effect on changing transporter expression is minimal on transmission at a single synapse. Therefore, it is unlikely to be functionally important unless the data reflect a large change in a limited set of neurons. Obviously, the authors do not know that and furthermore have not perturbed vglut expression to assess its role in behavior. Thus, this vglut result is too preliminary and its current consideration weakens the presentation. Also, the authors may be making a mistake in their reasoning: implication of per suggests that the clock running by changes in transcription factors is needed, but it does not imply that the changes in glutamate reflect a change in gene expression. Rather, it may be that sensory inputs act independently of gene expression to interact with the gene expression-dependent clock neurons to change behavior. In this view, their search for a change in gene expression to explain the effect on glutamate release may be misguided. Overall, there are some interesting results here, particularly with respect to implicating glutamate release in rhythmic behavior. But the consideration of the data should be more cautious. Regarding the vglut data, it should be relegated to the supplemental data or not included in this manuscript because this aspect is too preliminary and not convincingly linked to the main findings. Likewise, the suggestion that it underlies the changes in glutamate should be removed form the figure and deemphasized in the text.

\# We are grateful for the helpful suggestion. As reviewer mentioned, there was a statistical difference in *vglut* mRNA expression, but the difference was not drastic. We understand the reviewer's concern that such small differences in *vglut* expression alone cannot cause the photoperiodic change in brain glutamate levels, and our previous text on this result was an over-suggestion. Thus, according to the reviewers’ and editors’ suggestion, we transferred the results of qPCR (previous Fig.8) to supplemental data, and removed *vglut* expression texts from the abstract and result, and revised the discussion in ***L320-334*** and conceptual diagram (new Figure 8). Thank you very much for all the advice you have given us to improve our research.

Reviewer #2

In this revised manuscript, the authors have addressed my major concerns directly through new experiments and observations or by providing additional information supporting their rationale and interpretation of results.

In summary: In response to the concern that they had not established the extent to which glutamate collected from the culture media reflected "release," the authors provide strong evidence that it does, using TTX to inhibit AP firing and showing a significant reduction on glutamate. In response to the concern that differences in glutamate levels between photoperiods might simply reflect a changed phase in glutamate abundance rhythms, the authors have added data for an additional diurnal timepoint. In response to the concern that the knockdown of Period might be incomplete and therefore not lead to a loss of circadian timekeeping, the authors state that the same experimental manipulation stops the circadian rhythm in cuticle deposition.

The authors have also provided clear responses to the minor concerns voiced in my critique of the original manuscript. The authors have therefore done an admirable job responding to these concerns and I have no further issues with the work, which is a very nice contribution.

\# We are very grateful for kind comments to our research. Thank you very much for all the advice you have given us to improve our research.

Reviewer #3

I applaud the authors for addressing my previous comments and conducting additional experiments to strengthen the manuscript. The addition of the vglut expressions data (that it changes with photoperiod based on new mRNA data) is exciting. However, in looking over the newly added model figure (which is a great addition), there is a clear disconnect; specifically the authors did not report any data linking period gene function to changes in vglut expression in long vs short day conditions. Given that the authors can target period for knock down using RNAi, I would suggest the authors examine the expression of vglut in the presence or absence of dsper in long vs short day conditions.

\# We are grateful for the helpful suggestion. We agree with the reviewer’s suggestion that there is a disconnection regarding the relationship between *period* and *vglut* expression. Although it is possible to analyze the effect of RNAi-mediated *period* knockdown on *vglut* expression, other reviewer is concerned about discussing the importance of VGLUT in glutamate dynamics solely from the qPCR result; there was a statistical difference in *vglut* mRNA expression, but the difference was not so drastic. Thus, according to the editors’ and other reviewer’s suggestion, we transferred the results of qPCR (previous Fig.8) to supplemental data, and removed the abstract and result texts about *vglut* expression, and toned down the discussion in ***L320-334***, and revised the conceptual diagram (new Figure 8). Thank you very much for all the advice you have given us to improve our research.

Note to editor and reviewers

　# In Fig.1, we previously used parametric statistical analyses (Welch-Aspin test, Tukey-Kramer test). On the other hand, we accurately analyzed the data distribution by Shapiro-Wilk test, and found that some data are not normally distributed. Because nonparametric analyses are more suitable for these non-normally distributed data, we tested again with nonparametric tests (Mann–Whitney *U* test, Steel–Dwass test) and obtained statistical results similar to the parametric tests. We revised the figure legend texts of the statistical method in Fig. 1 ***(L895, L897***) and added the explanation that the normality of the data was tested by Shapiro-Wilk test to the Material and Method section (***L627-631***). We would like to note that this re-test did not affect the statistical results in Fig.1 (just only the P value in Fig1C became more significant from P <0.01 to P <0.001).
